# Correlated impulses: Using Facebook interests to improve predictions of crime rates in urban areas

**DOI:** 10.1371/journal.pone.0211350

**Published:** 2019-02-04

**Authors:** Masoomali Fatehkia, Dan O’Brien, Ingmar Weber

**Affiliations:** 1 Princeton University, Princeton, NJ, United States of America; 2 School of Public Policy and Urban Affairs and School of Criminology and Criminal Justice, Northeastern University, Boston, MA, United States of America; 3 Boston Area Research Initiative, Northeastern and Harvard Universities, Boston, MA, United States of America; 4 Qatar Computing Research Institute at Hamad Bin Khalifa University, Doha, Qatar; Stony Brook University, Graduate Program in Public Health, UNITED STATES

## Abstract

Much research has examined how crime rates vary across urban neighborhoods, focusing particularly on community-level demographic and social characteristics. A parallel line of work has treated crime at the individual level as an expression of certain behavioral patterns (e.g., impulsivity). Little work has considered, however, whether the prevalence of such behavioral patterns in a neighborhood might be predictive of local crime, in large part because such measures are hard to come by and often subjective. The Facebook Advertising API offers a special opportunity to examine this question as it provides an extensive list of “interests” that can be tabulated at various geographic scales. Here we conduct an analysis of the association between the prevalence of interests among the Facebook population of a ZIP code and the local rate of assaults, burglaries, and robberies across 9 highly populated cities in the US. We fit various regression models to predict crime rates as a function of the Facebook and census demographic variables. In general, models using the variables for the interests of the whole adult population on Facebook perform better than those using data on specific demographic groups (such as Males 18-34). In terms of predictive performance, models combining Facebook data with demographic data generally have lower error rates than models using only demographic data. We find that interests associated with media consumption and mating competition are predictive of crime rates above and beyond demographic factors. We discuss how this might integrate with existing criminological theory.

## Introduction

Urban criminologists have long sought to understand variations in crime across communities within a city. Most of this work has focused on ecological theories of criminogenesis and the contextual factors that encourage and discourage crime. Early work highlighted the elevated levels of crime in areas of high poverty and concentrated disadvantage (e.g. [1]). One of the most influential theories in the study of crime in communities has been social disorganization theory, which posits that social ties and the ability to establish and enforce social norms are critical to socializing residents and managing local spaces [2–4]. Others have argued that income inequality instigates those lower on the socioeconomic spectrum to turn to crime for self-advancement [5].

Paralleling ecological theories for community-level variations in crime, there are numerous theories regarding the characteristics of individual residents that incline them toward crime. One prominent example is the self-control, or “general”, theory of crime [6] and its offshoots (e.g., the general theory of deterrence [7]), which focuses on the role of impulsivity in driving criminal behavior. Another is general strain theory, which argues that negative experiences, or “strains”, can lead to emotions that sometimes motivate criminal behavior [8]. In a separate corner of academia, evolutionary psychologists have contended that the mating context is an important factor for understanding crime. In particular, they argue that violence, property crime, and other acts that could be seen as directly or indirectly conferring status are most motivated by the perceived need to compete for mates, especially for young males (e.g. [9, 10]). Such theories have occasionally been tested with urban populations, but most often because they offer a study site with a readily available sample (e.g. [11]) or a particular context that lends itself to testing the theory (e.g., homelessness and strain theory [12]). In some cases there is also the desire to test both individual- and community-level hypotheses simultaneously, though generally independently of each other [13–15]. In a few rare cases researchers have also examined whether the neighborhood context interacts with individual-level characteristics, amplifying or mitigating their effects on criminal behavior [16].

Here we ask a distinct question that remains unanswered in this work: could the spatial distribution of individual-level processes further explain the variation in crime between neighborhoods? One of the challenges of such an exercise is the need for comprehensive measures of the behaviors and attitudes of residents. Such information would typically be cost-prohibitive to collect, but the advent of social media has generated such data for millions of people across the world. Here we leverage the Facebook Advertising API, which enables users to search an extensive list of “interests” that can be tabulated at various geographic scales. Using the Facebook Advertising API, we conduct an analysis of the association between the prevalence of interests among the Facebook population of a ZIP code, which is typically the lowest feasible geographical unit for tabulation in the API, and the local rate of assaults, burglaries, and robberies across nine highly populated cities in the US. We treat the interests searched through the Facebook Advertising API as expressions of particular aspects of behavior that are implicated by individual-level theories of crime (i.e., manifestations of latent behavioral constructs). For example, a preference for gambling or card games might be an indicator of impulsivity, whereas a preference for crosswords or classical music requires a patience that might indicate a lack thereof. We then assess whether the nature of these interests in a given ZIP code are predictive of the three types of crime rates over and above demographic measures derived from census data.

Before proceeding to the methods and analysis, it is important to state a few caveats. First, this study is exploratory, thus we propose no a priori hypotheses linking particular preferences to a given theory of criminality. Such a step would be premature as this study is the first to our knowledge to use preferences to reflect behavioral and attitudinal features of a population. Instead, we take the corpus of preferences as a whole and undertake the initial task of examining its factor structure and use the resultant factors to predict crime rates. In the Discussion we return to the possible linkages with previous theory, anticipating that this will provide a baseline for future work that tests theoretically-driven hypotheses. Second, the results here should be interpreted precisely as the prediction of reported crime rates, not necessarily crime itself. Though such reports largely reflect actual rates of crime, there is reason to believe that they are influenced by policing strategies that may be more likely to target certain populations or to result in arrests and thus crime reports in certain communities [17, 18]. This will need to be kept in mind when interpreting the results.

## Materials and methods

### Data

The study utilized three data sources describing ZIP codes in nine U.S. cities: (i) “interests” from the Facebook Advertising API; (ii) demographic measures according to the U.S. Census; and (iii) crime events accessed from each city’s public data portal. Data collection was performed in compliance with the terms of services of the websites from which data was collected. The nine cities–Baltimore, Boston, Chicago, Dallas, Los Angeles, New York, Philadelphia, San Francisco and Washington–were selected as all of the cities among the 25 largest by population in the 2010 U.S. Census that made crime incident data available in a way that could be tabulated for ZIP codes. The analysis that follows is limited to ZIP codes with a population of at least 10,000 in the 2015 American Community Survey (ACS) and where the ratio of the Facebook users estimates to 2015 population was less than 1.5. This limits to locations where we can feel confident that Facebook’s systems for determining home are accurate, while also permitting some leeway for population shifts or dynamics that may lead to more Facebook users being identified living in a place than the ACS would have estimated (e.g., neighborhoods with colleges and universities). In the end, 432 ZIP codes fitting these criteria were included in the analysis.

#### Facebook advertising API

Facebook enables potential advertisers to target a desired audience defined through one or more characteristics, including geographic location, demographics (e.g., gender and age), behavior and interests. Some of the user attributes available for targeting are based on self-reported information, like demographics, while others are based on online activity on Facebook and Facebook-affiliated services, while yet others are provided by third party data brokers. Facebook’s advertising platform called Ad Manager provides advertisers with an estimate of the number of Monthly Active Users (MAUs) matching the specified targeting criteria. This information is typically used by advertisers to plan their campaign budget. However, it is also useful for research purposes as it acts as a “digital census” across Facebook’s user population allowing aggregate, anonymous data to be collected regarding characteristics of the user population.

The Facebook data were collected from Facebook’s Ads Manager through Facebook’s Graph API. For each ZIP code in the dataset, data was collected on two classes of Facebook interests: those describing patterns of media consumption; and measures of relationships and dating.

For the Facebook interests, estimates of number of users with each interest were tabulated for each ZIP code. We did this for the entire population, and then repeated after segmenting the population by gender and age groups (18-24, 18-34, 25+, 18+) and all combinations thereof. We did this because certain demographic groups are more likely to be perpetrators or victims (e.g., a large percentage of crime is committed by males from 18-24), and we sought to examine whether the interests of one of these population segments was more predictive of crime rates. To illustrate, the API estimated that there are 840 Facebook users living in ZIP code 11221 (in Brooklyn, New York City) who are aged 18-24, female and interested in classical music. We then calculated the fraction of users from the given demographic group with various interests as follows:

$$\begin{array}{c} \text{Fraction}\;\text{of}\;\text{users}\;\text{with}\;\text{interest}\;\text{X}=\frac{\text{Estimated}\;\text{users}\;\text{with}\;\text{interest}\;\text{X}}{\text{Estimated}\;\text{users}\;\text{in}\;\text{the}\;\text{demographic}\;\text{group}} \end{array}$$ (1)

To continue the previous example, ZIP code 11221 has approximately 10,000 females aged 18-24, generating a final fraction of .084 or 8.4%. Note that the Facebook advertising API typically produces round numbers for total estimates of Facebook users.

For data on relationship statuses, aggregate user estimates in each ZIP code for the various combinations of age, gender and relationship statuses were collected, including “interest in an online dating service”. Again, we collected these for the entire adult population and for age-and-gender-defined segments and computed their prevalence using Eq (1). Next, for each demographic group, the female to male ratio of the users in each relationship status was computed and used in the subsequent analysis. The female to male ratio of those in an “open relationship” status was excluded due to data sparsity. We collected this ratio as we hypothesized that a gender imbalance in openly declaring to be in a relationship could be tied to risk-taking behavior.

The Facebook data was collected over the period October-November 2017. For this project we complied with the terms of service of Facebook’s Marketing API (https://developers.facebook.com/policy/#marketingapi). In particular, any data collected was anonymous (15. 8.b) and we did not build or augment any user profiles (15. 8.f). The data collection was done using pySocialWatcher, a library based on the python programming language which automates the data collection process by sending queries through Facebook’s API for the specified criteria [19].

#### Demographic variables

For each of the 432 ZIP codes, data on demographic composition was collected from the 2011-2015 five-year estimates provided by the American Community Survey (ACS) [20]. The demographic data captured details on age, income, racial composition and educational attainment for the adult population for each ZIP code.

Importantly, the U.S. Census approximates ZIP codes with ZIP Code Tabulation Areas (ZCTA) [21], whereas Facebook’s Advertising API uses true ZIP codes. ZIP codes and ZCTAs are nearly identical, with the rare exceptions typically including non-residential public buildings such as offices and schools situated along ZIP code boundaries which may fall into the ZCTA of an adjacent ZIP code area. Public parks will often not be included in any ZCTA as they have no residents. Given their similarity, we treat ZIP codes and ZCTAs interchangeably in the analysis.

#### Crime rates

National crime reporting systems such as FBI’s Uniform Crime Reporting system (UCR) do not report US wide crime data at the ZIP code level. As a result, the crime data used in this study were compiled from datasets released by individual city agencies who reported such data at the required geographic granularity (i.e. ZIP code level), working down the list of US cities in descending order of population. While the national crime reporting system in the US includes a broad list of crimes organized into various categories, our analysis here focuses on three types of crimes, namely assaults, burglaries and robberies. These crimes are among the most common offenses thus ensuring adequate coverage across the various cities’ datasets and ZIP codes, thereby mitigating data sparsity issues given the small geographic granularity. The crime data collection process involved the following steps:

1. We acquired geocoded crime incident data from the open data portals of the respective cities [22–30];
2. We then spatially joined each crime incident to its containing ZIP code (using the ‘sp’, ‘rgeos’, ‘rgdal’ and ‘tmap’ packages in R; over 99% of crime incidents could be mapped this way);
3. In order to ensure comparable definitions of crime types across cities, we mapped the crime categories used in each individual city’s dataset to the standardized categorization used by the National Incident Based Reporting System (NIBRS) [31].
4. We calculated crime rates per 100,000 residents by tabulating the number of crime events in a given category by ZIP code and then dividing by the ACS 2015 population estimate and multiplying by 100,000.

#### Preliminary analysis for variable selection

As noted, Facebook interests for each ZIP code were collected not only for the whole population but for specific age groups, genders, and their combinations. We did this because it is well-established that certain demographic groups (e.g., males 18-24) are more likely to be involved in crime than others, in which case their interests might be more predictive of crime rates. As the activities and interests of different demographic groups correlate differently with crime, it is possible that the Facebook interests and relationship variables for certain demographic groups may be more strongly predictive of crime rates than others.

We used linear predictive models in order to evaluate the efficacy of Facebook interests in predicting crime rates across ZIP codes in the data set based on the interests of each of the various demographic groups. The results were then evaluated using a cross validation approach to provide estimates of out of sample predictive performance of the models. Given the large number of Facebook variables, the linear model were fitted using the LASSO model fitting technique which selects only a subset of the variables for the model [32].

See S1 Table in the appendix for a summary of the performance of the various models. It is evident that data from the 18+ demographic group had the strongest predictive fit overall, attaining the lowest error. Based on this observation, for the rest of the analysis done here we use the data collected for this demographic group only.

### Measures

Before analysis we conducted a series of factor analyses to reduce the large number of Facebook interests and demographic measures down to a more manageable and interpretable set of dimensions [33].

Prior to factor analysis, we separated our Facebook-derived measures into four groupings: (i) movie related interests, (ii) music related interests, (iii) video and online game related interests and (iv) relationship and dating related variables. We then conducted a factor analysis within each grouping. We also conducted a fifth factor analysis of demographic variables. The number of factors to compute was decided based on the additional variance explained by the extra factor. Factor analysis was carried out using the ‘factanal’ package in the R programming language. Since this package performs factor analysis using the correlation matrix of the variables, variables were not standardized beforehand. Once the factor loadings were computed, variables whose loading’s absolute value exceeded 0.4 were marked as contributing to that factor and used to compute the factor scores for that factor in each ZIP code [34]. We then named the factors to descriptively capture their content. In all cases the variables that loaded together were sufficiently similar to be able to create such a label. Please refer to Tables 1 and 2 for factors and loadings of the groupings of Facebook variables. We report the same results for the demographic data in the S2 Table as it is a more standard analysis and less pertinent to our main goals here. S1 Appendix describes the factor analysis and the process of computing the factor scores in some more details.

**Table 1:** Factor analysis of the Facebook interest related variables.

Games	F1	F2	Music	F1	F2	F3	Movies	F1	F2
Games	0.811	0.408	Music	0.402	0.317	0.486	Movies	0.599	0.474
Word	0.021	0.329	Music videos	0.871	0.046	0.225	Animated	0.686	0.233
Action	0.525	0.816	Classical	-0.096	0.160	0.540	Comedy	0.766	0.107
Gambling	0.530	0.399	Gospel	0.943	0.148	-0.092	Fantasy	0.730	0.110
Online poker	0.076	-0.339	Soul	0.942	0.135	0.070	Science fiction	0.597	0.606
First-person shooter	0.811	0.140	Jazz	0.742	0.109	0.186	Thriller	0.836	0.266
Online	0.661	0.496	Dance	0.750	0.008	0.319	Action	0.902	0.120
Simulation	0.678	0.417	Rhythm & blues	0.975	0.176	0.045	Musical theatre	-0.020	0.473
Casino	0.416	0.709	Hip hop	0.962	-0.027	0.092	Documentary	0.383	0.882
Board	0.250	-0.027	Blues	0.249	0.915	0.143	Drama	0.577	0.248
Role-playing	0.627	0.043	Country	-0.227	0.914	0.070	Anime	0.567	-0.335
Racing	0.693	0.266	Rock	0.281	0.698	0.472	Horror	0.838	0.362
Browser	0.319	0.588	Heavy metal	0.193	0.772	0.442			
Shooter	0.768	0.195	Electronic	0.405	0.216	0.779			
Sports	0.707	0.379							
Strategy	0.456	0.393							
Card	0.330	0.746							
Puzzle video	0.411	0.907							
Video games	0.836	0.410							

Factors and their loadings on the various Facebook interest related variables for the categories of games, music and movies.

**Table 2:** Factor analysis of the Facebook relationship related variables.

Relationship	F1	F2	F3
female/male ratio with “dating” status	0.980	0.136	0.126
female/male ratio with “single” status	0.741	0.401	0.142
female/male ratio with “in a relationship” status	0.967	0.112	0.095
female/male ratio with “unspecified” status	0.255	0.954	-0.143
Fraction of males “in a relationship” w. interest in “Online dating service”	0.005	-0.107	0.436
Fraction of females “in a relationship” w. interest in “Online dating service”	0.143	0.047	0.514

Columns indicate the factors and their loadings on the various Facebook relationship variables.

The factor analyses generated a series of dimensions that we then used as independent variables in the proceeding analysis. There were three dimensions of music interests that we might refer to as: *hip-hop, soul and related*; *rock-country*; and *easy listening*. There was only one independent dimension related to movies (a second was overly correlated with the first factor) that encompassed interest in comedy, fantasy, thriller, action, drama, and horror movies. There was also only one independent dimension related to games (a second was overly correlated with the first factor), which encompassed, action, online, casino, browser, sports, card, and puzzle games. There were three dimensions related to relationships and dating: *female-to-male ratios across stated statuses*; *female-to-male ratio in “unspecified” status*; and *fraction of females “in a relationship” with an interest in online dating services*. There were three dimensions of demographics: *affluence*; *proportion Black*; and *concentrated disadvantage*.

### Analysis

Once the factor scores were computed, they were used as the input variables in Ordinary Least Squares (OLS) regression models seeking to explain the geographic variation in crime rates for each of the three crimes of assaults, robberies and burglaries. Three different OLS models were fit each using a different subset of variables: (i) the factor scores from the demographic data only, (ii) the factor scores from the Facebook variables only, (iii) the factors scores from both the demographic variables and the Facebook variables.

**Fig 1:**
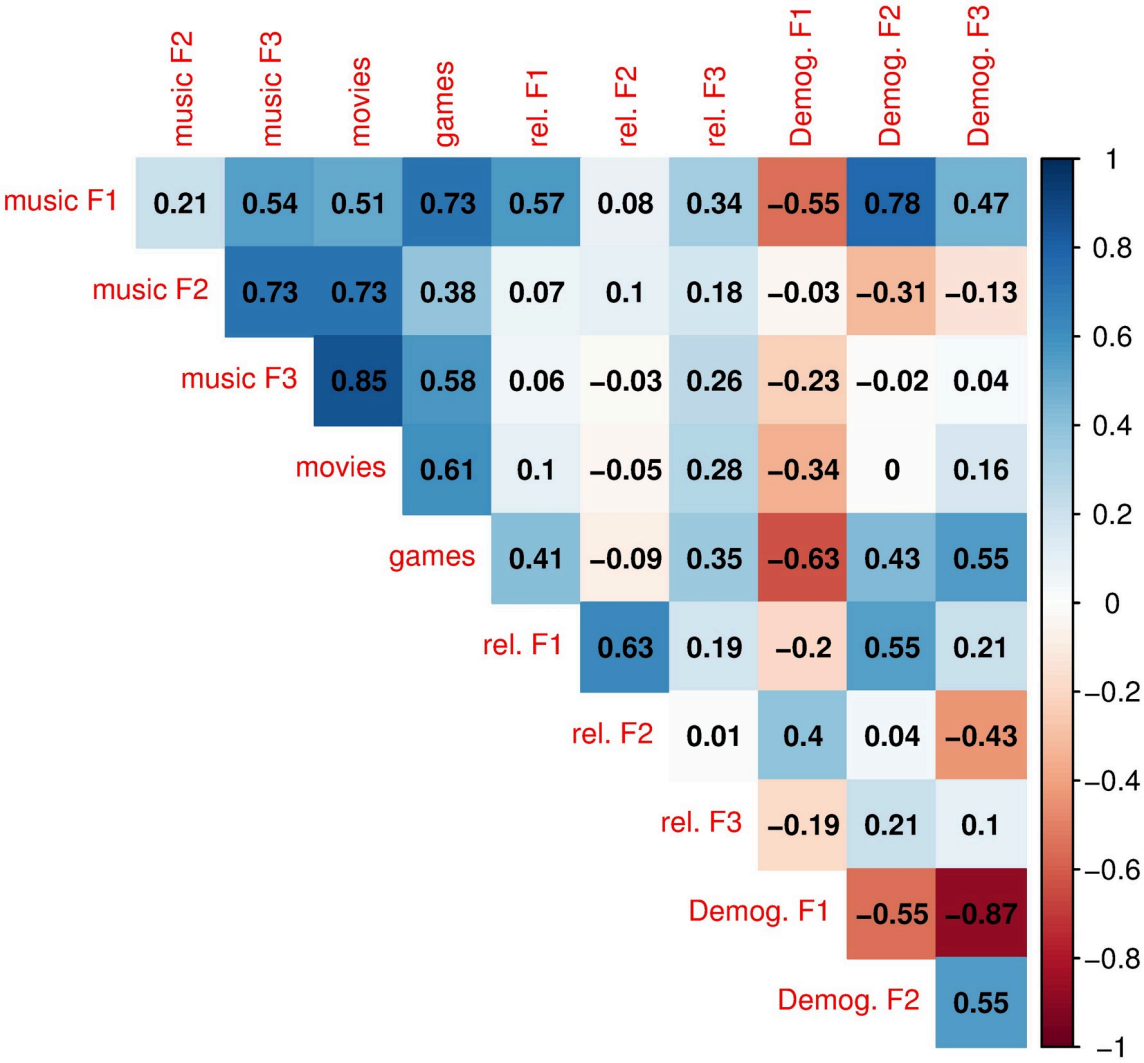
Correlation matrix of Facebook and demographic factor variables.

Models were fit with a backwards regression procedure in which we iteratively removed the least significant variables at each step until all model variables were significant at the 5% p-value. This was necessary given heavy collinearity between variables. Fig 1 shows the correlations between the various Facebook and demographic factors.

For the final set of models chosen through the step-wise regression procedures, we report two measures of model performance: (i) the adjusted R-squared, (ii) Mean Absolute Error (MAE). We report the MAE of the models on the dataset as well as an estimate of out-of-sample predictive error of the models computed through a cross validation approach; the cross validation involved fitting the chosen model on different subsets of the dataset and averaging its error over the subsets it was not fitted on.

## Results

We ran a series of three regression models for each of the three crime types–assaults, burglaries, and robberies. The details of the regression models are presented in Table 3 for assaults, Table 4 for burglaries and Table 5 for robberies. The first model used only demographic factors to predict crime rates, the second used only Facebook interests, and the third used both. All models included a series of dummy variables controlling for each city’s baseline crime rate and also controlled for age composition of the neighborhood (percentage of 18-24 year-olds and median age), which might be correlated both with crime rates and with Facebook usage patterns. Owing to collinearity between variables, the models were run as a backwards stepwise regression, beginning with a comprehensive model and then trimming back to only the significant predictors. Across all three, we see that the combination of demographic factors and Facebook interests had the greatest predictive strength for both the initial in-sample (using adjusted *R*^2^) and the out-of-sample prediction (using *MAE*), though the advantage over the demographics-only model varied by crime type.

**Table 3:** Parameter estimates and fit statistics for regression models predicting assault crimes.

	Demog. Model	FB Model	Demog. & FB Model
*Intercept*	-0.100 (0.123)	-0.187 (0.131)	-0.016 (0.123)
*% Population aged 18-24*	-0.075 (0.042)		-0.021 (0.043)
*Median age*	-0.208*** (0.040)		-0.129** (0.045)
*affluence*			
*proportion black*	0.553*** (0.031)		0.262** (0.080)
*concentrated disadvantage*			0.220*** (0.052)
*hip-hop soul and related*		0.590*** (0.035)	0.332*** (0.097)
*rock-country*		-0.528*** (0.052)	
*easy listening*			
*movies factor*			-0.143* (0.061)
*games factor*			-0.170** (0.064)
*female-to-male ratios across stated statuses*			
*female-to-male ratio in “unspecified” status*			
*frac. Of females “in a relationship” with an interest in online dating services*			
Adjusted R-squared	0.639	0.604	0.656
Marginal adjusted R-squared (from city dummies)	0.488	0.437	0.511
RSS	151.50	166.78	143.17
MAE (train)	437.77	450.89	423.50
MAE (CV)	450.18	465.38	439.08
F-statistics	70.44	66.70	55.76
df	420	421	416
N	432	432	432

*** p < 0.001,

** p < 0.01,

* p < 0.05

Demog. = demographic, FB = Facebook. Coefficient standard errors are in parenthesis. All variables were standardized before the regression. City-level fixed effects are accounted for by models.

**Table 4:** Parameter estimates and fit statistics for regression models predicting burglary crimes.

	Demog. Model	FB Model	Demog. & FB Model
*Intercept*	0.780*** (0.135)	0.922*** (0.137)	0.899*** (0.136)
*% Population aged 18-24*	-0.086 (0.047)		-0.039 (0.046)
*Median age*	-0.071 (0.044)		-0.023 (0.044)
*affluence*			
*proportion black*	0.199*** (0.035)		0.144* (0.059)
*concentrated disadvantage*			
*hip-hop soul and related*		0.524*** (0.057)	0.338*** (0.076)
*rock-country*			
*easy listening*			
*movies factor*		-0.152** (0.054)	
*games factor*		-0.249*** (0.054)	-0.275*** (0.052)
*female-to-male ratios across stated statuses*		-0.174*** (0.043)	-0.149*** (0.044)
*female-to-male ratio in “unspecified” status*			
*frac. Of females “in a relationship” with an interest in online dating services*			
Adjusted R-squared	0.562	0.601	0.598
Marginal adjusted R-squared (from city dummies)	0.083	0.163	0.157
RSS	183.77	167.22	167.62
MAE (train)	168.14	161.68	161.72
MAE (CV)	174.29	167.62	168.12
F-statistics	51.37	55.08	46.80
df	420	419	417
N	432	432	432

*** p < 0.001,

** p < 0.01,

* p < 0.05

Demog. = demographic, FB = Facebook. Coefficient standard errors are in parenthesis. All variables were standardized before the regression. City-level fixed effects are accounted for by models.

**Table 5:** Parameter estimates and fit statistics for regression models predicting robbery crimes.

	Demog. Model	FB Model	Demog. & FB Model
*Intercept*	0.761*** (0.136)	0.820*** (0.147)	0.906*** (0.137)
*% Population aged 18-24*	-0.119* (0.047)		-0.077 (0.047)
*Median age*	-0.292*** (0.044)		-0.272*** (0.046)
*affluence*			-0.170** (0.054)
*proportion black*	0.482*** (0.035)		0.250** (0.089)
*concentrated disadvantage*			
*hip-hop soul and related*		0.6*** (0.046)	0.283** (0.105)
*rock-country*		-0.645*** (0.059)	
*easy listening*			
*movies factor*			-0.240*** (0.070)
*games factor*			-0.171** (0.064)
*female-to-male ratios across stated statuses*		-0.139** (0.045)	
*female-to-male ratio in “unspecified” status*			
*frac. Of females “in a relationship” with an interest in online dating services*			
Adjusted R-squared	0.558	0.528	0.581
Marginal adjusted R-squared (from city dummies)	0.411	0.371	0.441
RSS	185.63	198.17	174.46
MAE (train)	111.61	116.16	109.85
MAE (CV)	115.18	120.32	114.55
F-statistics	50.47	44.86	40.78
df	420	420	416
N	432	432	432

*** p < 0.001,

** p < 0.01,

* p < 0.05

Demog. = demographic, FB = Facebook. Coefficient standard errors are in parenthesis. All variables were standardized before the regression. City-level fixed effects are accounted for by models.

As seen in Table 4, accounting for each city’s baseline crime rate, burglary rate was very weakly predicted by demographics (marginal *R*^2^ = .08; *MAE* = 174.3), but this rose with the incorporation of Facebook interests (marginal *R*^2^ = .16; *MAE* = 168.12). In fact, the model including only Facebook interests was not only much stronger than the demographics-only model, it was more efficient at capturing the same quantity of information as the combined model (*R*^2^ = .16; *MAE* = 167.62). In contrast, for assaults and robberies, demographics played a stronger role in predicting crime rates. In each case, the Facebook interests model was comparable in strength to the demographics-only model, though somewhat weaker (assaults, Table 3: marginal *R*^2^ = .44 vs .49, *MAE* = 465.35 vs. 450.18; robberies, Table 5: *R*^2^ = .37 vs .41, *MAE* = 120.32 vs. 115.18). That said, the combination of the two sets of predictors was always the most effective at predicting crime rates (assaults, Table 3: *R*^2^ = .51, increase in variance explained with the introduction of Facebook interests, *F* = 6.05, *p* <.001, *MAE* = 439.08; robberies, Table 5: *R*^2^ = .44, increase in variance explained with the introduction of Facebook interests, *F* = 7.53, *p* <.001, *MAE* = 114.55).

Examining the individual predictors more closely, we see some patterns across crime types, especially assault and robbery. Proportion Black predicted higher levels of assault, robbery, and burglary, with all effects remaining in the final models with Facebook interests included (assault, Table 3: *B* = .26, *p* <.001; burglary, Table 4: *B* = .14, *p* <.05; robbery, Table 5: *B* = .25, *p* <.01). Additionally, concentrated disadvantage independently predicted a higher assault rate (Table 3: *B* = .22, *p* <.001), though was unrelated to the other two outcomes in the final models. Concentrated poverty was not independently predictive of any of the three types of crime.

Results regarding Facebook interests were diverse, and shifted substantially between the Facebook interests-only and Facebook-and-demographics models. For this reason we describe the results for music interests, other media, and dating and relationships in turn. All three Facebook interests-only models found that hip-hop and soul predicted higher crime rates (assault, Table 3: *B* = .59, *p* <.001; burglary, Table 4: *B* = .52, *p* <.001; robbery, Table 5: *B* = .60, *p* <.001). In contrast, country-rock predicted lower assault and robbery rates (assault, Table 3: *B* = −.53, *p* <.001; robbery, Table 5: *B* = −.64, *p* <.001). These effects were largely attenuated by the incorporation of demographic factors, but not entirely. Country-rock was no longer predictive of any outcomes in the final models, but hip-hop and soul remained predictive of higher rates of all three types of crime, albeit to a lesser extent than in the Facebook interests-only models (assault, Table 3: *B* = .33, *p* <.001; burglary, Table 4: *B* = .34, *p* <.001; robbery, Table 5: *B* = .28, *p* <.01). The third factor based on music interests, easy listening, was not predictive of crime rates in any of the models.

Other media consumption had an inconsistent relationship with crime outcomes. Areas that had a greater density of people with interests in movies had lower burglary rates in the Facebook interests-only model (Table 4: *B* = −.15, *p* <.05), but this relationship was no longer present when demographics were incorporated. However, interests in movies *became* predictive of fewer assaults and robberies after accounting for demographics, despite not being a significant predictor in the Facebook interests-only model (assault, Table 3: *B* = −.14, *p* <.05; robbery, Table 5: *B* = −.24, *p* <.001). Similarly, interests in games (e.g., puzzles, video games, sports games) were associated with lower burglary rates in both models (Table 4, final model: *B* = −.28, *p* <.001), but only emerged as predictive of lower assault and robbery rates in once demographics were accounted for (assault, Table 3: *B* = −.17, *p* <.05; robbery, Table 5: *B* = −.17, *p* <.001).

In terms of dating and relationships, the ratio of females to males with specified dating statuses (e.g., in a relationship, single) predicted lower burglary and robbery rates in the Facebook interests-only models (burglary, Table 4: *B* = −.17, *p* <.001; robbery, Table 5: *B* = −.14, *p* <.01). It only remained a significant predictor, though, for burglary rates (Table 4, *B* = .15, *p* <.001). Neither of the other factors describing dating and relationships–ratio of females to males with an unspecified status and fraction of females “in a relationship” with an interest in online dating–was predictive of any of the three crime rates.

**Fig 2:**
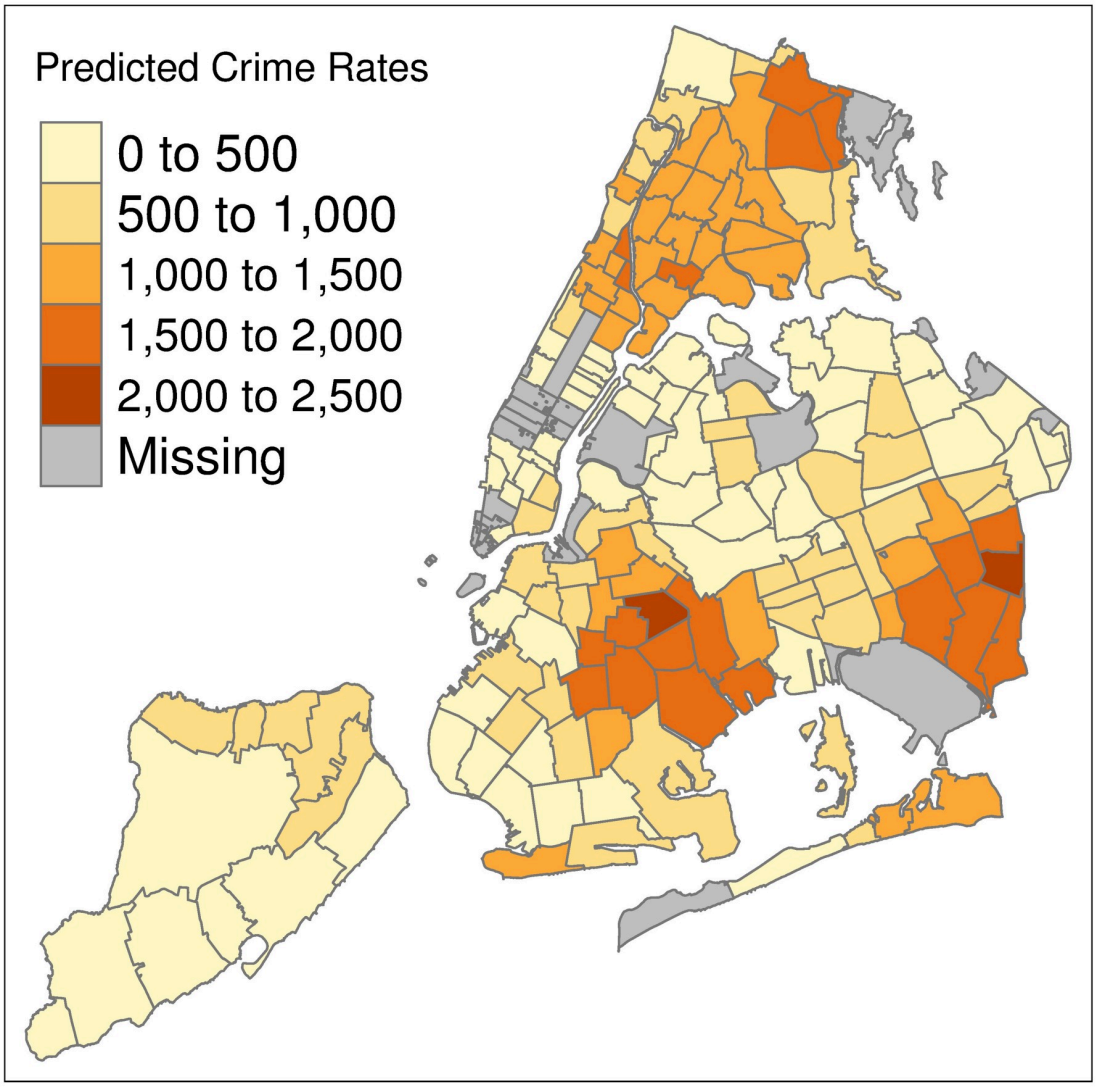
Predicted assault crime rates. Assault crime rates predicted by the Facebook model for ZIP codes in New York City.

**Fig 3:**
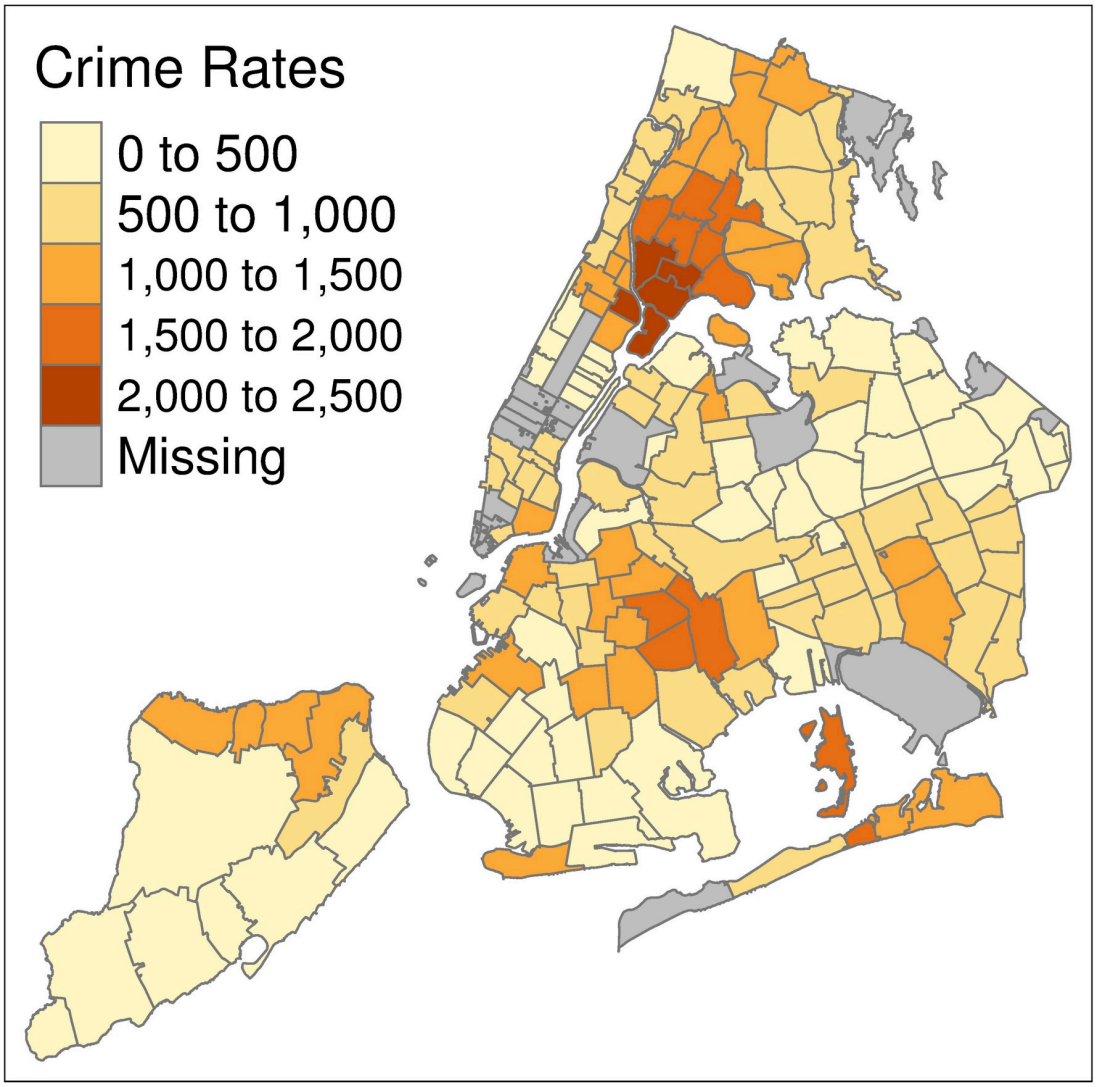
Assault crime rates 2017. 2017 Assault crime rates for ZIP codes in New York City.

As a visual illustration for New York city, Figs 2 and 3 show maps of the assault crime rates as predicted by the Facebook model and as reported in the year 2017 respectively. We choose to illustrate New York city as it is the most highly populated city in the dataset with a large coverage of ZIP code areas and relatively good model performance (prediction MAE of 325 for FB assault crime model). From this visual comparison, several observations can be made. For low crime areas, such as in Staten Island (bottom left), Brooklyn and Queens (bottom right) we generally observe predictions to be consistent with the reported crime rates; however, we also observe low crime areas that are predicted to have much higher crime rates (dark orange areas in bottom of the plot). We also observe lower predicted crime rates for the high crime areas in the Bronx/Manhattan (top right).

## Discussion

The results demonstrate the utility of Facebook interests to predict crime rates across the ZIP codes of a city. Across three types of crime—assaults, robberies, and burglaries—the prevalence of certain interests among the Facebook population improved the predictive power of models already accounting for the racial composition and socioeconomic status of the population. We also segmented the population by age and gender to see if interests among populations known to be more active as offenders (e.g., young males) were more strongly associated with crime rates, but Facebook interests of the overall population were the most effective predictors. This could be in part because the whole population will be substantially larger than any sub-population, permitting more precise measurement. Nonetheless, it suggests that the power of Facebook interests to predict crime rates lies less in the behaviors of particular individuals and more in the overall behavioral ecology of a region.

For assaults and robberies, demographics already explained a substantial proportion of the variance, but the Facebook interests offered an incremental improvement in model fit. Importantly, the models using Facebook interests but not demographics were only modestly less effective than models with demographics alone, indicating a strong set of intercorrelations between demographics, Facebook interests, and crime rates. The potential value of Facebook interests as a novel data set, however, shone through in the analysis of burglary rates. Burglary was only modestly explained by racial composition and socioeconomic status, as has been seen previously in the literature [35]. This is attributed to two contrasting pressures: more affluent neighborhoods offer more targets, but perpetrators are more likely to be lower income and tend to commit crimes closer to home [36]. In this regard, Facebook interests explained 16 percent of the marginal variance in predictions of burglary rates, which was twice that of the demographics-only model.

Just as important as the ability of Facebook interests to help predict future crime rates across ZIP codes is how they do so. We saw measures from two generalized categories—media consumption and relationships and dating—each contribute to the models, though each appears to tell its own story. For media consumption, we see two sets of results. For music, a prevalence of people with interests in hip hop and soul and related genres predicted higher crime rates, even when controlling for racial composition and socioeconomic status. There are multiple possible explanations for this. First, it is possible that the poor black neighborhoods hit hardest by high crime rates are those where hip hop music and related genres are most popular. In fact, this finding might also be driven in part by a decoupling of race and culture, where neighborhoods dominated by other ethnicities, by they White, Latino, Asian, or otherwise, that listen to large amounts of hip hop and related music also have elevated crime rates. Alternatively, it could be that neighborhoods with cultural interests that are stereotypically associated with crime might be monitored more strongly by police, resulting in more crimes identified and arrests (e.g., [37]).

On the other hand, elevated interests in movies predicted lower rates of assault and robbery controlling for demographics, and interests in games predicted lower rates of burglary. This combination of findings might point to a potential pattern in that those items that predicted fewer crimes are reflective of a more subdued lifestyle with a greater investment of time in the consumption of digital media. What this means theoretically, however, might be debated. For example, proponents of the general theory of crime might argue that such consumption is a manifestations of a more patient, future-oriented perspective on life that also is reflected in lower crime rates. Strain theory might instead rephrase the same distinction, viewing these activities as a potential outlet for strain, which would not then be channeled into crime. A third, simpler interpretation would be that people who are spending large amounts of time watching movies and playing video have fewer opportunities to commit crimes as they are occupied with these activities.

Relationships and dating were also associated with crime, though only in that a higher ratio of females with stated relationship statuses to males with stated relationship status predicted burglary rates. It is difficult to extrapolate too much from this single finding, though it is consistent with one theoretical perspective in this area that focuses on male-male intrasexual competition. It may be that the greater overall availability of females in a community–as indicated by the high ratio of females to males reporting any sort of dating status– leads males to be less likely to commit crime as they have less need to compete for attention. That said, it is not entirely clear whether this is because of availability of females for particular types of relationships, or whether this is just a broader impact of male-female sex ratio, which is well-captured by “ratio of females to males with a stated status” as most individuals indicate their status [9].

As noted at the forefront, this study was exploratory, seeking to determine whether Facebook interests could act as a novel tool that could improve upon demographic-driven models for predicting crime rates across the neighborhoods of a city. We found, indeed, that this is the case, and thus believe there are opportunities for constructing more formalized forecasting systems using these resources, especially if the work is expanded to more cities than the 9 we were able to examine here. There is also the potential to use these data to test competing and complementary theories of criminogenesis at the neighborhood and individual levels [38]. That said, there are important limitations that will need to be addressed moving forward. The first entails the precision of the methodology. Here we have taken an omnibus approach to testing the relationship between a wide array of Facebook interests and crime rates. This is an appropriate first step, but will need to be followed by a more scrutinous approach that organizes interests according to theoretical propositions [39]. The Facebook Advertising API is also complex in its ability to segment the population by various demographic characteristics. This might be used to better specify “offender” and “victim” groups and how their own interests might be reflected. We have used this capacity limitedly here, finding that interests among young males were not more predictive of crime rates than interests of the general public. That said, there may be additional opportunities in this vein that could be probed. A challenge, however, will be data sparsity, as such segmentation reduces the overall pool of users from which data is collected.

Last, there is a general caveat for modern digital data resources: “researcher beware” [40–42]. The Facebook algorithms that generate the data are a black box, creating two issues. The first is one of substantive interpretation and what exactly it means for a particular ZIP code to be high in a given interest. The second is that changes in these algorithms would mean that the models need to be re-calibrated over time for long-term prediction purposes. We have demonstrated an initial set of relationships between the analyzed interests, demographics, and crime rates, but these only tell us so much about the data and even less about their stability in the coming years. More targeted work will need to be done to fully understand these data and their utility.

## Supporting information

S1 Table Preliminary analysis across various demographic groups on Facebook. (PDF)

S2 Table Factor loadings for the demographic variables. (PDF)

S1 Appendix Details on the factor analysis. (PDF)

## References

[1] Booth C. Life and Labour of the People in London. London: Macmillan & co.; 1903.

[2] Shaw C, McKay H. Juvenile Delinquency and Urban Areas. Chicago: University of Chicago Press; 1942/1969.

[3] Bursik RJ, Grasmick H. Neighborhoods and Crime: The Dimensions of Effective Community Control. New York: Lexington Books; 1993.

[4] Sampson RJ. Great American City: Chicago and the Enduring Neighborhood Effect. Chicago: University of Chicago Press; 2012.

[5] Wilson M, Daly M. Life expectancy, economic inequality, homicide, and reproductive timing in Chicago neighbourhoods. British Medical Journal. 1997;314:1271–1274. 10.1136/bmj.314.7089.1271 9154035 PMC2126620

[6] Gottfredson MR, Hirschi T. A General Theory of Crime. New York, NY: Macmillan; 1990.

[7] Nagin DS, Pogarsky G. Integrating celerity, impulsivity, and extralegal sanction threats into a model of general deterrence: Theory and evidence. Criminology. 2001;39(4):865–892. 10.1111/j.1745-9125.2001.tb00943.x

[8] Agnew R. Foundation for a general strain theory of crime and delinquency. Criminology. 1992;30(1):47–87.

[9] Kanazawa S, Still MC. Why Men Commit Crimes (and Why They Desist), Why Men Commit Crimes (and Why They Desist). Sociological Theory. 2000;18(3):434–447. 10.1111/0735-2751.00110

[10] Buss DM. The Evolutionary Psychology of Crime. Journal of Theoretical and Philosophical criminology. 2012;1(1):90–98.

[11] Aseltine J, Robert H, Gore S, Gordon J. Life stress, anger and anxiety, and delinquency: An empirical test of general strain theory. Journal of Health and Social Behavior. 2000;41(3):256–275. 10.2307/2676320 11011504

[12] Baron SW. General strain theory, street youth, and crime: A test of Agnew’s revised theory. Criminology. 2004;42(2):457–483. 10.1111/j.1745-9125.2004.tb00526.x

[13] O’Brien DT, Kauffman RA. Broken windows and low adolescent prosociality: Not cause and consequence but co-symptoms of low collective efficacy. American Journal of Community Psychology. 2013;51(3-4):359–369. 10.1007/s10464-012-9555-1 23180238

[14] Sampson RJ, Morenoff JD, Earls F. Beyond Social Capital: Spatial Dynamics of Collective Efficacy for Children. American Sociological Review. 1999;64(5):633–660. 10.2307/2657367

[15] Chase-Lansdale PL, Gordon RA. Economic hardship and the development of five- and six-year-olds: Neighborhood and regional perspectives. Child Development. 1996;67(6):3338–3367. 10.2307/1131782

[16] Lynam DR, Caspi A, Moffitt TE, Wikstrom POH, Rolf L, Novak S. The interaction between inpulsivity and neighborhood context on offending: The effects of impulsivity are stronger in poorer neighborhoods. Journal of Abnormal Psychology. 2000;109(4):563–574. 10.1037/0021-843X.109.4.563 11195980

[17] Harcourt BE. Illusion of Order: The False Promise of Broken Windows Policing. Cambridge, MA: Harvard University Press; 2001.

[18] Taylor RB. Breaking away from broken windows: Baltimore neighborhoods and the nationwide fight against crime, grime, fear, and decline. Westview Press Boulder, CO; 2001.

[19] Araujo M, Mejova Y, Weber I, Benevenuto F. Using Facebook Ads Audiences for Global Lifestyle Disease Surveillance: Promises and Limitations. In: Proceedings of the 2017 ACM on Web Science Conference. WebSci’17. New York, NY, USA: ACM; 2017. p. 253–257. Available from: http://doi.acm.org/10.1145/3091478.3091513.

[20] US Census Bureau. 2012-2016 American Community Survey 5-Year Estimates; 2015. Available from: https://factfinder.census.gov/faces/nav/jsf/pages/searchresults.xhtml?refresh=t.

[21] US Census Bureau. ZIP Code Tabulation Areas (ZCTAs); 2015. Available from: https://www.census.gov/geo/reference/zctas.html.

[22] New York Police Department. NYPD Complaint Data Historic; 2018. Available from: https://data.cityofnewyork.us/Public-Safety/NYPD-Complaint-Data-Historic/qgea-i56i.

[23] Boston Police Department. Crime Incident Reports (August 2015—To Date); 2018. Available from: https://data.boston.gov/dataset/crime-incident-reports-august-2015-to-date-source-new-system/resource/12cb3883-56f5-47de-afa5-3b1cf61b257b.

[24] Chicago Police Department. Crimes—2001 to present | City of Chicago | Data Portal; 2018. Available from: https://data.cityofchicago.org/Public-Safety/Crimes-2001-to-present/ijzp-q8t2.

[25] Los Angeles Police Department. Crime Data from 2010 to Present; 2018. Available from: https://data.lacity.org/A-Safe-City/Crime-Data-from-2010-to-Present/y8tr-7khq.

[26] Baltimore Police Department. crime | Open Baltimore | City of Baltimore’s Open Data Catalog; 2018. Available from: https://data.baltimorecity.gov/Crime/crime/rp9g-42r9.

[27] District of Columbia Metropolitan Police Department. Crime Incidents in 2017; 2018. Available from: http://opendata.dc.gov/datasets/6af5cb8dc38e4bcbac8168b27ee104aa_38?page=2803.

[28] San Francisco Police Department. Police Department Incidents; 2018. Available from: https://data.sfgov.org/Public-Safety/-Change-Notice-Police-Department-Incidents/tmnf-yvry.

[29] Dallas Police Department. Police Incidents; 2018. Available from: https://www.dallasopendata.com/Public-Safety/Police-Incidents/tbnj-w5hb.

[30] Philadelphia Police Department. Crime Incidents; 2018. Available from: https://www.opendataphilly.org/dataset/crime-incidents.

[31] Law Enforcement Support Section Crime Statistics Management Unit. National Incident-Based Reporting System (NIBRS) User Manual; 2013. https://ucr.fbi.gov/nibrs/nibrs-user-manual.

[32] Tibshirani R. Regression Shrinkage and Selection via the Lasso. Journal of the Royal Statistical Society Series B (Methodological). 1996;58(1):267–288. 10.1111/j.2517-6161.1996.tb02080.x

[33] Comrey AL, Lee HB. A First Course in Factor Analysis. Psychology Press; 2013.

[34] Tabachnick BG, Fidell LS. Using Multivariate Statistics. 5th ed New York: Allyn and Bacon; 2006.

[35] Liu H, Zhu X. Exploring the influence of neighborhood characteristics on burglary risks: A Bayesian random effects modeling approach. ISPRS International Journal of Geo-Information. 2016;5(7):102 10.3390/ijgi5070102

[36] Townsley M, Birks D, Bernasco W, Ruiter S, Johnson SD, White G, et al Burglar Target Selection: A Cross-national Comparison. Journal of Research in Crime and Delinquency. 2015;52(1):3–31. 10.1177/0022427814541447 25866418 PMC4361488

[37] Rios VM. Punished: Policing the lives of Black and Latino boys. NYU Press; 2011.

[38] Messner SF, Rosenfeld R. Crime and the American dream. Cengage Learning; 2012.

[39] O’Brien DT, Sampson RJ. Public and private spheres of neighborhood disorder: Assessing pathways to violence using large-scale digital records. Journal of research in crime and delinquency. 2015;52(4):486–510. 10.1177/0022427815577835

[40] Boyd D, Crawford K. Critical questions for big data: Provocations for a cultural, technological, and scholarly phenomenon. Information, communication & society. 2012;15(5):662–679. 10.1080/1369118X.2012.678878

[41] Lazer D, Kennedy R, King G, Vespignani A. The parable of Google Flu: traps in big data analysis. Science. 2014;343(6176):1203–1205. 10.1126/science.1248506 24626916

[42] Kitchin R. Big Data, new epistemologies and paradigm shifts. Big Data & Society. 2014;1(1):2053951714528481.

