# Supplementary material for: Correlated impulses: Using Facebook interests to improve predictions of crime rates in urban areas
Source: PLoS One. 2019 Feb 4;14(2):e0211350. doi: 10.1371/journal.pone.0211350 (PMC6361434; doi:10.1371/journal.pone.0211350)
Supplement: S1 Table — (PDF) [file pone.0211350.s001.pdf]

**S1 Table. Preliminary analysis across various demographic groups on Facebook**

| | | Assault | | | Burglary | | | Robbery | | |
|---|---|---|---|---|---|---|---|---|---|---|
| | **Demog. Group** | **size** | **Train** | **CV** | **size** | **Train** | **CV** | **size** | **Train** | **CV** |
| 1 | All aged 18+ | 19 | 455.53 | 461.27 | 7 | 233.04 | 204.94 | 13 | 121.06 | 118.72 |
| 2 | Male aged 18+ | 15 | 529.87 | 538.88 | 6 | 264.15 | 232.56 | 23 | 120.96 | 124.48 |
| 3 | Female aged 18+ | 16 | 500.21 | 487.87 | 6 | 240.68 | 213.27 | 10 | 130.49 | 129.74 |
| 4 | Male aged 18-34 | 18 | 549.80 | 554.84 | 5 | 284.05 | 256.39 | 16 | 130.82 | 131.54 |

The errors reported here are the Mean Absolute Error (MAE). Errors report the error on the dataset (train error) as well as an estimate of predictive error of the model on unseen observations computed using a cross validation approach (CV error). Demog. stands for demographic and size indicates the number of variables used in the model.
