# Supplementary material for: Correlated impulses: Using Facebook interests to improve predictions of crime rates in urban areas
Source: PLoS One. 2019 Feb 4;14(2):e0211350. doi: 10.1371/journal.pone.0211350 (PMC6361434; doi:10.1371/journal.pone.0211350)
Supplement: S2 Table — (PDF) [file pone.0211350.s002.pdf]

S2 Table. Factor loadings for the demographic variables

Demographic variables	**F1**	**F2**	**F3**
% of Population aged 15-19	-0.233	0.182	0.161
% Households income < 25K	-0.534	0.416	0.446
% Households income > 150K	0.922	-0.259	-0.265
Median family income	0.848	-0.246	-0.359
% Population Black/African-American	-0.199	0.977	-0.018
% Population White	0.299	-0.752	-0.318
% Population 25+ less than high school	-0.485	0.082	0.868
% Population 25+ w. Bachelors degree or higher	0.728	-0.308	-0.457

The columns indicate the factors and their loadings on each of the demographic variables.
