# Supplementary material for: Correlated impulses: Using Facebook interests to improve predictions of crime rates in urban areas
Source: PLoS One. 2019 Feb 4;14(2):e0211350. doi: 10.1371/journal.pone.0211350 (PMC6361434; doi:10.1371/journal.pone.0211350)
Supplement: S1 Appendix — (PDF) [file pone.0211350.s003.pdf]

S1 Appendix. Details on the factor analysis

Factor analysis attempts to explain a group of observed variables in a dataset by a smaller set of underlying factors. This can be expressed as:

$$x_{ik} = \sum_{j=1}^{m} l_{ij} * F_{jk} + \epsilon_{ik}$$

x_{ik} is the value of the ith variable for the kth observation, eg: the fraction of Facebook users with interests in classical music in a given ZIP code. l_{ij} are factor loadings which indicate the weights on the factors when computing the value of the ith variable. Finally ϵ_{ik} are error terms.

The factors loadings l_{ij} were computed using the 'factanal' package in the R programming language. Using the computed factor loadings and after determining the number of factors to keep, the values of the individual factors scores F_{jk} were computed one by one as follows:

- The variables that were considered as loading on the jth factor were identified; these were variables whose factor loading was greater than 0.4.
- After determining the variables loading onto the factor, the value of the factor score for each ZIP code was computed as:

  $$F_{jk} = \frac{\sum_i l_{ij} * x_{ik}}{\sum_i l_{ij}^2}$$

  where the sums in the numerator and denominator are over the variables that were identified as contributing to the jth factor.
